# The CEASE Tobacco Cessation Controlled Trial for Low-Income Racial and Ethnic Minority Participants: Key Predictors of Success

**DOI:** 10.31586/gjcd.2025.1246

**Published:** 2025-02-19

**Authors:** Shervin Assari, Rifath Ara Alam Barsha, Chidubem Egboluche, Payam Sheikhattari

**Affiliations:** 1 Charles R Drew University of Medicine and Science, Los Angeles, CA, USA; 2 University of Mississippi Medical Center, MS, USA; 3 Morgan State University, Baltimore, MD, USA

**Keywords:** Tobacco, Quit, Trial, Intervention, Ethnic Groups

## Abstract

**Background:**

Tobacco use remains disproportionately high among low-income and racial-ethnic minority populations. The CEASE program, with its self-help, hybrid/online, and in-person modalities, has demonstrated efficacy in promoting tobacco cessation. However, predictors of successful cessation among participants in these groups remain unclear.

**Objective:**

To identify baseline predictors of successful tobacco cessation among low-income and racial-ethnic minority participants in the CEASE program, with a focus on demographic, socioeconomic, behavioral, and psychosocial factors.

**Methods:**

Participants were allocated into three intervention arms: self-help, CEASE hybrid/online, and CEASE in-person. Baseline characteristics, including demographics (e.g., age, gender), socioeconomic status (e.g., education, employment), substance use profiles (e.g., cigarette packs per week, use of other tobacco products, menthol tobacco use), physical health (e.g., general health, number of cardiometabolic risk conditions), mental health (e.g., depressive symptoms, perceived stress), perceived social support, and nicotine dependence, were analyzed as potential predictors of cessation success. Multivariable logistic regression models were used to identify factors associated with successful quitting, controlling for the study arm.

**Results:**

In addition to the study arm, gender, baseline depression, cardiometabolic conditions, tobacco flavor, and the use of other tobacco products were significant predictors of quit success. Individuals receiving in-person interventions had significantly higher odds of quitting (AOR = 3.79, *p* < 0.05). Women were significantly less likely to quit compared to men (AOR = 0.24, *p* < 0.01). Participants with a greater number of cardiometabolic risk conditions were more likely to quit (AOR = 1.93, *p* < 0.05), while those with higher levels of depression had lower odds of quitting (AOR = 0.61, *p* < 0.05). Menthol tobacco users were also less likely to quit (AOR = 0.10, *p* < 0.05). Interestingly, individuals who used other forms of tobacco in addition to cigarettes had increased odds of quitting (AOR = 2.86, *p* < 0.05). No other factors, including demographic variables (e.g., age), socioeconomic status (e.g., education, marital status), substance use profiles (e.g., cigarette packs per week, NRT use), or nicotine dependence, were significant predictors of cessation success.

**Conclusion:**

Baseline self-reported anxiety/depression and depressive symptoms play a critical role in reducing the likelihood of successful tobacco cessation among low-income and racial-ethnic minority participants in the CEASE program. These findings underscore the importance of addressing mental health challenges as part of tobacco cessation interventions to enhance their efficacy. Future research should explore targeted strategies for integrating mental health support into cessation programs to improve outcomes for underserved populations.

## 1. Introduction

Although tobacco use has declined significantly over the past several decades in the United States [1], this overall progress masks a persistent disparity [2–5]: Tobacco use remains disproportionately high among individuals from low-income and racial-ethnic minority populations [6,7]. These groups face unique barriers to quitting, including limited access to resources, targeted marketing by the tobacco industry, and higher exposure to stressors such as financial insecurity and systemic inequities [8–10]. As a result, tobacco dependence has become increasingly concentrated among these populations, contributing to widening tobacco health disparities [4].

Despite the significant burden of tobacco use in low-income and minority communities, relatively few evidence-based cessation programs have been designed specifically for these populations [11]. Among the existing interventions, the Communities Engaged and Advocating for a Smoke-Free Environment (CEASE) program has demonstrated efficacy in promoting tobacco cessation among low-income and minority individuals [12–17]. CEASE is a community-based participatory research (CBPR) initiative and peer-facilitated smoking cessation program. Developed as a CBPR project in Baltimore, CEASE has evolved through iterative refinements to address the unique needs of underserved populations. The program incorporates tailored counseling, nicotine replacement therapy (NRT), and culturally sensitive support to ensure its relevance and effectiveness. Its success highlights its potential as a promising approach to reducing tobacco use and addressing related health disparities in underserved communities [12–17].

CEASE is a community-based participatory research (CBPR) initiative and peer-facilitated smoking cessation program tailored to address the multifaceted needs of underserved populations. Developed and iteratively refined in Baltimore, CEASE incorporates a comprehensive approach by addressing key factors such as age, gender, education, and employment status. The program also considers participants’ general health, the number of cardiometabolic risk conditions, levels of depression, perceived stress, and perceived social support, ensuring that interventions are contextually relevant. In addition to tailored counseling, CEASE includes nicotine replacement therapy (NRT) and culturally sensitive support, with specific attention to cigarette packs per week, other tobacco product use, and baseline nicotine addiction levels [18–22]. These refinements position CEASE as a highly promising approach to reducing tobacco use and related health disparities within underserved communities [12–17].

This study aims to identify the key predictors of successful tobacco cessation within the CEASE program among low-income and racial-ethnic minority populations. By examining a range of constructs, including demographics, socioeconomic status, substance use profiles, physical and mental health, nicotine dependence, and social support, this research seeks to provide actionable insights that can enhance the design and delivery of tobacco cessation interventions for underserved populations.

## 2. Methods

### 2.1. Study Design and Setting

This study utilized a randomized cluster trial design, in which three Baltimore City communities were randomly assigned to one of three study arms: (1) in-person intervention, (2) hybrid/virtual intervention, and (3) self-help/control group. To ensure randomization was unbiased, the allocation process was blinded and conducted during a steering committee meeting. The communities selected—Oldtown/Middle East, Waverly, and Southwest (including Poppleton, The Terraces, Hollins Market, Washington Village, and Pigtown)—were chosen for their comparable sociodemographic characteristics and shared access to schools, faith-based organizations, and healthcare facilities. Initially, the program included a fully virtual intervention arm, but due to logistical challenges, this arm transitioned to a hybrid format. Recruitment efforts required expanding the geographic boundaries of the communities, and participants outside these initial boundaries were randomized by site [15].

### 2.2. Ethical Considerations

The study received ethical approval from Morgan State University’s Institutional Review Board (IRB #19/06-0082). Informed consent was obtained from all participants before their enrollment in the study. Participant confidentiality was protected by assigning unique identification numbers, which were stored separately from contact information.

### 2.3. Study Participants

Eligibility criteria for the CEASE Digital smoking cessation program included: (1) being at least 21 years old, (2) currently smoking three or more cigarettes per day, (3) expressing willingness and readiness to quit smoking, and (4) providing informed consent. Participants in the fully virtual intervention arm needed reliable access to internet-enabled devices, such as desktops, laptops, or tablets. This requirement was waived for hybrid sessions conducted in person. Individuals unable to provide consent due to health conditions were excluded [15].

### 2.4. Intervention and Study Arms

The smoking cessation program was facilitated by nine trained peer motivators, some of whom were former smokers with at least one year of abstinence, while others had personal connections to tobacco use through family or close relationships. Peer motivators underwent a three-day workshop covering the CEASE Digital curriculum, digital platform integration, research ethics, data management, and group facilitation. They also participated in recruitment efforts and served as primary facilitators for the sessions [15].

Recruitment strategies included community outreach, word-of-mouth referrals, flyers, social media campaigns, and community surveys. Smoking cessation classes were held at community locations such as public housing sites, senior centers, churches, and other venues. Recruitment occurred between April 2022 and September 2023.

The CEASE Digital smoking cessation program was developed in collaboration with community stakeholders and experienced facilitators. The curriculum spanned seven weeks for participants in the in-person and hybrid/virtual arms. The in-person program used the CEASE Today Tobacco Cessation Manual alongside printed cessation materials. The hybrid/virtual program featured an online platform with modules designed to mirror the in-person curriculum. Sessions for the hybrid/virtual arm were conducted via Zoom, and printed materials were distributed as needed.

The program was structured as follows: Week 1 focused on informed consent and distribution of orientation materials; Week 2 provided technology training for Zoom sessions; and Weeks 3 to 7 included five two-hour weekly sessions covering motivation, preparation for quitting, active cessation, and relapse prevention. Participants in the self-help/control group received a one-hour motivational session, self-help materials (both physical and digital), and information about local cessation resources.

### 2.5. Questionnaires

Baseline questionnaires captured data on socio-demographics, smoking behaviors, physical and mental health, social support, and related variables. Follow-up surveys were administered approximately three months after intervention completion for in-person and hybrid/virtual participants and five months after enrollment for the self-help/control group.

### 2.6. Measures

#### Outcome Variable:

The primary outcome was smoking status at follow-up, categorized as either quit (1) or not quit (0).

#### Predictors:

Key predictors included age, gender, race, intervention arm, education, employment, general health, number of cardiometabolic risk conditions, nicotine dependence (measured by the Fagerström Test for Nicotine Dependence), other tobacco use, flavored tobacco use, number of weekly cigarette packs consumed, depression, perceived stress, and perceived social support.

#### Number of Cardiometabolic Conditions:

Participants self-reported chronic medical conditions, including high blood pressure, heart attack or other heart diseases, diabetes, and obesity. These conditions were summed to calculate the total number of cardiometabolic risk conditions, resulting in a score ranging from 0 to 4.

#### Depressive Symptoms:

Measured using the Patient Health Questionnaire-2 (PHQ-2), with a Cronbach’s alpha of 0.88. This measure was treated as a continuous variable. A higher score indicated higher depressive symptoms.

#### Perceived Social Support:

Assessed using the Duke/UNC Functional Social Support Questionnaire, with a Cronbach’s alpha of 0.91. This measure was treated as a continuous variable. A higher score indicated higher perceived social support.

#### Perceived Stress:

Measured with the Perceived Stress Scale-4 (PSS-4), with a Cronbach’s alpha of 0.51. This measure was treated as a continuous variable. A higher score indicated higher perceived stress.

### 2.7. Statistical Analysis

Univariate analyses summarized baseline characteristics, while bivariate analyses explored differences across study arms using Chi-square tests for categorical variables and Kruskal-Wallis tests for continuous variables. Logistic regression models (both unadjusted and adjusted) examined relationships between predictors and smoking cessation outcomes, reporting odds ratios (ORs), 95% confidence intervals (CIs), and p-values. A significance level of p < 0.05 was used. Data analysis was conducted using Stata 15.0 (StataCorp LLP).

## 3. Results

### 3.1. Descriptive Data and Bivariate Analysis

Table 1 presents the descriptive results of the study. The majority of participants were aged over 50 years (78.4%), women (50.5%), Black Americans (82.8%), and unemployed (88.4%). Menthol tobacco use was reported by 84.0% of participants. The mean (SD) nicotine addiction score at baseline was 4.4 (2.0). The mean (SD) scores for depression, perceived stress, and perceived social support were 1.3 (1.7), 5.3 (3.1), and 4.1 (1.0), respectively.

The two groups did not differ significantly in terms of education, employment status, nicotine addiction at baseline, number of cardiometabolic risk conditions, perceived stress, or social support. However, 45.6% of participants who received the in-person intervention reported quitting. In contrast, the majority of participants in the virtual/hybrid and self-help groups did not quit. Among those who quit, a majority were men (58.7%). Additionally, the mean (SD) scores for weekly cigarette packs consumed and depression were lower among those who quit—2.8 (2.2) and 0.8 (1.5), respectively.

### 3.2. Correlations

Table 2 presents the bivariate correlations among the study variables. Depression demonstrated a significant negative correlation with quitting smoking (r = −0.14, p < 0.05). No other variables were significantly correlated with quitting smoking.

### 3.3. Multivariable Analysis

The results of the multivariable logistic regression analysis are presented in Table 3. Individuals receiving in-person interventions demonstrated significantly higher odds of quitting (AOR = 3.79, p < 0.05). Women were significantly less likely to quit compared to men (AOR = 0.24, p < 0.01). Participants with a higher number of cardiometabolic risk conditions had increased odds of quitting (AOR = 1.93, p < 0.05), while individuals with higher levels of depression were less likely to quit (AOR = 0.61, p < 0.05). Menthol tobacco users were also less likely to quit (AOR = 0.10, p < 0.05). Conversely, those who used other forms of tobacco in addition to cigarettes were more likely to quit (AOR = 2.86, p < 0.05).

## 4. Discussion

This study sought to identify predictors of successful tobacco cessation among low-income and racial-ethnic minority participants in the CEASE program, focusing on three intervention modalities: self-help, hybrid/online, and in-person. In addition to the study arm, gender, baseline depression, cardiometabolic conditions, tobacco flavor, and the use of other tobacco products were significant predictors of quit success. Women were significantly less likely to quit compared to men (AOR = 0.24, p < 0.01). Participants with a higher number of cardiometabolic risk conditions had increased odds of quitting (AOR = 1.93, p < 0.05), while individuals with higher levels of depression were less likely to quit (AOR = 0.61, p < 0.05). Menthol tobacco users were also less likely to quit (AOR = 0.10, p < 0.05). Conversely, those who used other forms of tobacco in addition to cigarettes were more likely to quit (AOR = 2.86, p < 0.05). Notably, no other baseline factors, including demographics, socioeconomic status, physical health, or nicotine dependence, were predictive of cessation success, even after adjusting for intervention modality.

The identification of depressive symptoms as barriers to tobacco cessation aligns with existing literature that underscores the bidirectional relationship between mental health and tobacco use. Individuals with anxiety and depression often use tobacco as a maladaptive coping mechanism, creating a reinforcing cycle of dependency that complicates cessation efforts. Among low-income and racial-ethnic minority populations, this challenge may be exacerbated by higher levels of chronic stress and exposure to structural inequities, which not only elevate the prevalence of mental health conditions but also diminish access to mental health resources. Our findings suggest that addressing these mental health challenges is essential for improving the efficacy of tobacco cessation programs in these populations.

Interestingly, other factors commonly thought to influence cessation success, such as socioeconomic status and nicotine dependence, were not significant predictors in this study. This finding is particularly noteworthy given the extensive research linking these factors to cessation outcomes in the general population. One possible explanation is that the CEASE program, through its tailored interventions, effectively mitigates the influence of these factors by providing targeted support and resources. For example, access to nicotine replacement therapy (NRT) and culturally sensitive counseling may have reduced the impact of nicotine dependence, while programmatic flexibility in hybrid and in-person modalities may have addressed barriers related to socioeconomic constraints. However, the absence of significant effects for these variables underscores the complexity of cessation outcomes and the need for further research to elucidate the mechanisms at play.

These effects were net of the intervention arms—self-help, hybrid/online, and in-person CEASE. We found, as reported elsewhere [15], that in-person intervention predicts highest cessation success compared to other modes of the program. We need to incorporate mental health support into various CEASE formats. The CEASE program’s efficacy for low-income and minority populations makes it a valuable model for addressing health disparities in tobacco use. However, our findings also point to a critical gap in current intervention strategies: the lack of dedicated components to address mental health challenges as part of tobacco cessation efforts.

As shown before [23], cardiometabolic diseases did predict successful tobacco cessation in this study. Individuals with a higher number of chronic metabolic and cardiovascular illnesses may have greater motivation to quit smoking, given their heightened awareness of the health risks associated with continued tobacco use. Additionally, healthcare providers frequently counsel such patients with chronic conditions to quit smoking as part of their disease management plans. These factors should, in theory, enhance the likelihood of quitting. However, the presence of chronic disease alone may not be enough to drive cessation success in low-income and racial-ethnic minority populations. Systemic barriers, such as limited access to quality healthcare or culturally relevant cessation resources, may overshadow the motivating effects of chronic disease in these groups.

Similarly, it was surprising that social support did not emerge as a significant predictor of tobacco cessation success. Existing research consistently demonstrates that individuals with robust social support networks are more likely to succeed in behavioral change efforts, including smoking cessation [19,24]. Social support is often thought to provide emotional encouragement, accountability, and tangible assistance, all of which can facilitate quitting. However, in the context of low-income and racial-ethnic minority populations, the role of social support may be more complex. For instance, social networks in these communities may also include individuals who smoke, potentially undermining the positive effects of support. Alternatively, structural stressors and social inequities may limit the effectiveness of social support as a buffer against the challenges of quitting.

The lack of a significant relationship between socioeconomic status (SES) and cessation success was also unexpected but aligns with the theory of Minorities’ Diminished Returns (MDRs) [25,26]. According to MDRs, SES factors such as higher education and income yield weaker health-related benefits for racial and ethnic minority groups compared to their White counterparts [27–35]. In this context, higher SES may not translate to greater access to resources or opportunities that support quitting. Structural barriers, such as discrimination, stress, and inequities in healthcare access, likely mitigate the protective effects of SES in these populations. This finding underscores the importance of tailoring tobacco cessation interventions to address the unique challenges faced by low-income and minority individuals, rather than relying solely on SES as a marker of resilience or readiness to quit.

### Clinical Implications

From a clinical perspective, our findings emphasize the critical need to screen, diagnose, and treat anxiety and depression in individuals attempting to quit smoking or entering tobacco cessation programs like CEASE. Mental health conditions are not only common among tobacco users but also present significant barriers [24,36] to successful cessation, as highlighted by our study. Given the high comorbidity of tobacco use with other substance use and mental health conditions, integrating mental health support into cessation interventions is essential. This could include incorporating mental health screening tools, offering access to evidence-based treatments such as cognitive-behavioral therapy (CBT), and fostering collaboration between mental health providers and tobacco cessation counselors. By addressing the dual burden of tobacco dependence and mental health challenges, we can improve the effectiveness of interventions like CEASE and contribute to reducing health disparities in underserved populations.

### Public Health Implications

From a public health perspective, these results have important implications for the design and implementation of tobacco cessation programs in underserved populations. Integrating mental health screening and treatment into cessation interventions could significantly enhance outcomes by addressing one of the primary barriers to quitting. For example, incorporating evidence-based therapies, such as cognitive-behavioral therapy (CBT) for anxiety and depression, alongside traditional cessation tools like NRT, may offer a more comprehensive approach to supporting participants. Additionally, training program staff to recognize and address mental health symptoms, as well as fostering partnerships with mental health providers, could further strengthen the impact of these interventions.

### Limitations

While this study provides valuable insights, it is not without limitations. First, the reliance on self-reported measures for mental health and cessation outcomes may introduce bias, particularly among populations that face stigma related to mental health or tobacco use. Future research could benefit from incorporating objective measures, such as biochemical verification of cessation status and standardized clinical assessments of mental health. Second, our study focused exclusively on baseline characteristics, leaving room for future work to examine how changes in mental health status during the intervention period influence cessation outcomes. Finally, while our findings are generalizable to low-income and racial-ethnic minority populations, further research is needed to explore whether similar patterns emerge in other high-risk groups, such as individuals with co-occurring substance use disorders or those experiencing homelessness.

## 5. Conclusion

In conclusion, this study highlights the multifaceted predictors of tobacco cessation success in the CEASE program, including baseline depression, cardiometabolic risk conditions, gender, and the use of menthol or other tobacco products. The findings underscore the critical importance of addressing mental health challenges, such as depressive symptoms, alongside tobacco dependence to improve cessation outcomes, particularly among low-income and racial-ethnic minority participants. Additionally, the role of cardiometabolic health suggests that integrating broader health interventions may further support quit success. By prioritizing mental health, cardiometabolic health, and tailored interventions that consider gender and tobacco use patterns, cessation programs can more effectively reduce health disparities and foster equitable progress in tobacco control for underserved populations.

## Figures and Tables

**Table 1.** Descriptive Statistics of the Study Sample - Overall and by Quit Status

Variables	Quit Smoking		Overall (n=232)
	No (n=186)	Yes (n=46)	
Study Arm			
In-person	58 (31.2)	21 (45.6)	79 (34.0)
Virtual/Hybrid	74 (39.8)	16 (34.8)	90 (38.8)
Self-help	54 (29.0)	9 (19.6)	63 (27.2)
Age (years)			
50 years or less	39 (21.0)	8 (17.4)	47 (20.3)
More than 50 years	144 (77.4)	38 (82.6)	182 (78.4)
Missing	3 (1.6)	-	3 (1.3)
Gender			
Men	80 (43.0)	27 (58.7)	107 (46.1)
Women	98 (52.7)	19 (41.3)	117 (50.4)
Missing	8 (4.3)	-	8 (3.5)
Race			
Black American	151 (81.2)	41 (89.1)	192 (82.8)
White	23 (12.4)	4 (8.7)	27 (11.6)
Other/Multiple	7 (3.7)	1 (2.2)	8 (3.4)
Missing	5 (2.7)	-	5 (2.2)
Educational Attainment			
Some high school or less	53 (28.5)	11 (23.9)	64 (27.6)
Graduated from high school/GED	67 (36.0)	15 (32.6)	82 (35.3)
Some college	42 (22.6)	12 (26.1)	54 (23.3)
Bachelor or more	20 (10.8)	8 (17.4)	28 (12.1)
Missing	4 (2.1)	-	4 (1.7)
Employment			
No	162 (87.1)	43 (93.5)	205 (88.4)
Yes	17 (9.1)	3 (6.5)	20 (8.6)
Missing	7 (3.8)	-	7 (3.0)
Other Tobacco Product Use			
No	147 (79.0)	33 (71.7)	180 (77.6)
Yes	36 (19.4)	13 (28.3)	49 (21.1)
Missing	3 (1.6)	-	3 (1.3)
Flavored Tobacco Use			
Regular	11 (5.9)	7 (15.2)	18 (7.8)
Menthol	159 (85.5)	36 (78.3)	195 (84.0)
Other Flavor	2 (1.1)	-	2 (0.9)
Multiple Flavor	9 (4.8)	2 (4.3)	11 (4.7)
Missing	5 (2.7)	1 (2.2)	6 (2.6)
General Health			
Poor/Fair	91 (48.9)	24 (52.2)	115 (49.6)
Good/Excellent	91 (48.9)	22 (47.8)	113 (48.7)
Missing	4 (2.2)	-	4 (1.7)
	Mean (SD)	Mean (SD)	Mean (SD)
Nicotine Addiction (at Baseline)	4.1 (2.1)	4.2 (1.8)	4.4 (2.0)
Cigarette Packs per Week*	3.4 (2.5)	2.8 (2.2)	3.3 (2.4)
Number of Cardiometabolic Risk Conditions	1.0 (0.9)	1.3 (1.1)	1.1 (1.0)
Depression**	1.4 (1.7)	0.8 (1.5)	1.3 (1.7)
Perceived Stress	5.3 (3.1)	5.2 (3.0)	5.3 (3.1)
Perceived Social Support	4.0 (1.1)	4.3 (0.8)	4.1 (1.0)

Abbreviations: SD= Standard Deviation;

** P < 0.01,

* P < 0.05.

**Table 2.** Correlation Matrix of the Study Variables

	1	2	3	4	5	6	7	8	9	10	11	12	13
1.Quit Smoking	1.00												
2.Age	0.04	1.00											
3.Gender	−0.11	0.06	1.00										
4. Education	0.09	−0.01	0.05	1.00									
5.Employment	−0.04	−0.04	0.03	0.17**	1.00								
6.General Health	−0.02	−0.17*	−0.08	0.07	0.16*	1.00							
7. Number of Cardiometabolic Risk Conditions	0.10	0.28**	0.12	0.05	−0.03	−0.21**	1.00						
8.Depression	−0.14*	0.00	0.02	−0.03	−0.13*	−0.18*	0.21*	1.00					
9.Perceived Stress	−0.01	−0.17*	−0.01	−0.02	−0.01	−0.11	−0.07	0.42***	1.00				
10.Perceived Social Support	0.12	0.14*	0.17*	0.01	0.07	0.12	−0.01	−0.31***	−0.33***	1.00			
11. Cigarette Packs per Week	−0.11	−0.07	−0.03	0.04	0.01	−0.01	0.01	0.09	0.15*	−0.14*	1.00		
12.Oher tobacco Products Use	0.08	−0.17*	−0.08	−0.01	0.03	0.00	0.08	0.11	0.12	−0.13	0.08	1.00	
13.Nicotine Addiction (at Baseline)	−0.06	−0.01	−0.08	−0.18**	−0.17***	−0.05	−0.01	0.22***	0.17**	−0.16*	0.39***	−0.02	1.00

*** P < 0.001,

** P < 0.01,

* P < 0.05.

**Table 3.** Logistic Regression with Predictors of Quit

Variables	Quit Smoking	
	Unadjusted OR (95% CI)	Adjusted OR (95% CI)
Study Arm		
Self-help group	Ref.	Ref.
In-person group	2.17 (0.92, 5.16)	3.79* (1.07, 13.40)
Virtual/hybrid group	1.30 (0.53, 3.16)	1.78 (0.48, 6.58)
Race		
White	Ref.	Ref.
Black American	1.56 (0.51, 4.77)	2.04 (0.43, 9.64)
Others	0.82 (0.08, 8.60)	2.74 (0.14, 53.30)
Age		
50 years or less	Ref.	Ref.
More than 50 years	1.29 (0.56, 2.98)	1.39 (0.38, 5.03)
Gender		
Men	Ref.	Ref.
Women	0.57 (0.30, 1.11)	0.24** (0.09, 0.62)
Education		
Some high school or less	Ref.	Ref.
Graduated from high school/GED	1.08 (0.46, 2.54)	0.80 (0.25, 2.57)
Some college	1.38 (0.55, 3.43)	1.31 (0.37, 4.59)
Bachelor or more	1.93 (0.68, 5.49)	1.18 (0.27, 5.22)
Employment		
No	Ref.	Ref.
Yes	0.66 (0.19, 2.37)	0.73 (0.12, 4.29)
General Health		
Poor/Fair	Ref.	Ref.
Good/Excellent	0.92 (0.48, 1.75)	0.64 (0.25, 1.67)
Number of Cardiometabolic Risk Conditions	1.29 (0.91, 1.84)	1.93* (1.17, 3.19)
Depression	0.77* (0.61, 0.98)	0.61* (0.41, 0.90)
Perceived Stress	1.00 (0.90, 1.11)	1.06 (0.89, 1.27)
Perceived Social Support	1.39 (0.96, 2.01)	1.59 (0.90, 2.80)
Cigarette Packs per Week	0.87 (0.74, 1.03)	0.85 (0.66, 1.10)
Other Tobacco Product Use		
No	Ref.	Ref.
Yes	1.61 (0.77, 3.36)	2.86* (1.01, 8.09)
Flavored Tobacco Use		
Regular	Ref.	Ref.
Menthol	0.36* (0.13, 0.98)	0.10* (0.02, 0.58)
Other Flavors	-	-
Multiple Flavor	0.35 (0.06, 2.12)	0.14 (0.01, 1.55)
Nicotine Addiction (at Baseline)	0.93 (0.79, 1.09)	1.08 (0.84, 1.38)

Abbreviations: 0R= Odds Ratio; CI=Confidence Interval;

** P < 0.01,

* P < 0.05.
